# *PIM1* genetic alterations associated with distinct molecular profiles, phenotypes and drug responses in diffuse large B‐cell lymphoma

**DOI:** 10.1002/ctm2.808

**Published:** 2022-04-12

**Authors:** Huilai Zhang, Yaxiao Lu, Tingting Zhang, Qingpei Guan, Xiaoxuan Wang, Yixian Guo, Lanfang Li, Lihua Qiu, Zhengzi Qian, Shiyong Zhou, Wenchen Gong, Bin Meng, Xiubao Ren, Xianhuo Wang

**Affiliations:** ^1^ Department of Lymphoma Tianjin Medical University Cancer Institute and Hospital National Clinical Research Center of Cancer Key Laboratory of Cancer Prevention and Therapy Sino‐US Center for Lymphoma and Leukemia Research Tianjin China; ^2^ Marvel Medical Laboratory Tianjin Marvelbio Technology Co., Ltd Tianjin China; ^3^ Department of Pathology Tianjin Medical University Cancer Institute and Hospital Tianjin China; ^4^ Department of Immunology/Biotherapy Tianjin Medical University Cancer Institute and Hospital Tianjin China

Dear Editor,

Diffuse large B‐cell lymphoma (DLBCL) is a highly heterogeneous disease,^1^ and the high‐throughput sequencing has facilitated our understanding of genetic aberrations in DLBCL.^2^, ^3^, ^4^ The proviral integration site for Moloney murine leukemia virus 1 (*PIM1*), which encodes serine/threonine protein kinase, is identified as a target of aberrant somatic hypermutation in DLBCL^5^ and involved in tumorigenesis in hematopoietic malignancies^6^, ^7^ and solid cancers.^8^ Recent studies have revealed PIM1 mutation frequencies ranging from 20 to 30%.^9^, ^10^ However, there are few studies focused on its genetic alterations, molecular profiles, drug responses, and clinical significance. Here, we integrated targeted sequencing and transcriptome analysis to explore the pathogenic role of PIM1 mutations and as a personalized therapeutic target in *PIM1*‐mutated DLBCL patients.

A total of 188 patients underwent targeted sequencing using a 307 lymphoma‐related gene panel, and 162 patients were included in the analysis. A workflow chart is presented in Figure S1. Baseline characteristics are found in Table S1, and all variants identified are described in Table S2. See detailed methods in the Supporting Information. We found *PIM1* to be mutated in 46 (28.4%) patients (Figure 1A), with 164 genetic alterations (Table S3). Variant classifications showed that missense mutations occurred most frequently (84.1%); almost half of them (48.7%) are predicted to be deleterious (SIFT score < .05) (Figure 2A,B and Table S4). Besides, the C>T transition was the predominant type (54.4%) (Figure 2C). Of the 46 mutant patients, all samples harboured nonsynonymous alterations, with more than three mutations detected in a single sample from half of the patients (Figure 2D). We observed exon 4 to most often be mutated, and 57% (84/164) of mutations are located in the serine/threonine protein kinase domain (Figure 2E). Comutation and mutual exclusivity analysis identified 72 statistically significant interaction pair genes (Table S5 and Figure 2F), of which *PIM1* mutations significantly co‐occurred with *SETD1B* (*p *< .001), *CD79B* (*p *= .001) and MYD88 (*p *< .001) but not with *SPEN* mutations (*p *= .024) (Figure 2G). We also found that patients with *PIM1* mutations had higher mutation frequencies in *PRDM1* (*p *< .001) and *CD79B* (*p *= .001) involved in the NF‐κB pathway and B‐cell receptor pathway (Figure 2H). The important signalling pathway‐related genes are listed in Table S6.

**FIGURE 1:**
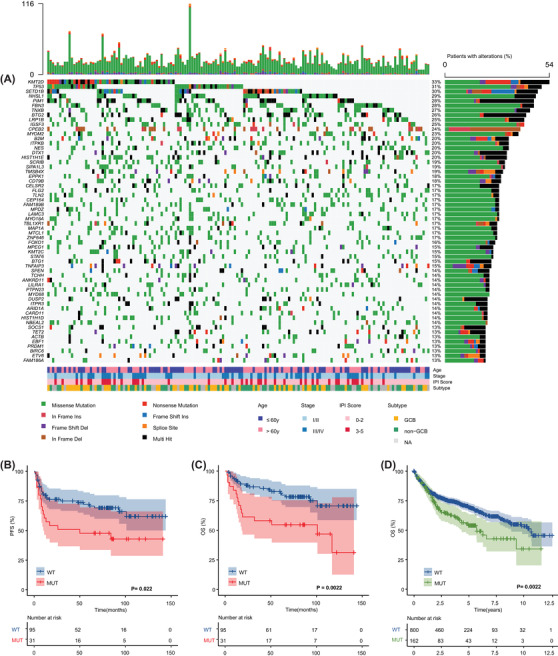
Genomic landscape and survival according to *PIM1* status in 162 diffuse large B‐cell lymphoma (DLBCL) patients. (A) The mutational heat map of top 60 high‐frequency mutation genes in 162 patients. Each row represents one gene and each column represents one patient. The bar at top represents the number of mutations a patient has. The vertical plot on the right depicts the number of mutations in each gene. Each mutation type is color coded as indicated by the legend. Clinical features, including age, stage, International Prognostic Index (IPI) score and GCB versus non‐GCB DLBCL subtype were provided. (B) The Kaplan–Meier survival curves of PFS for mutant and wild‐type *PIM1* patients (*p* = .022). (C) The Kaplan–Meier survival curves of overall survival (OS) for mutant and wild‐type *PIM1* patients (*p* = .0022). (D) The Kaplan–Meier survival curves of OS for mutant and wild‐type *PIM1* patients from publicly available data (*p* = .0022; accession number EGA: EGAS00001002606)

**FIGURE 2:**
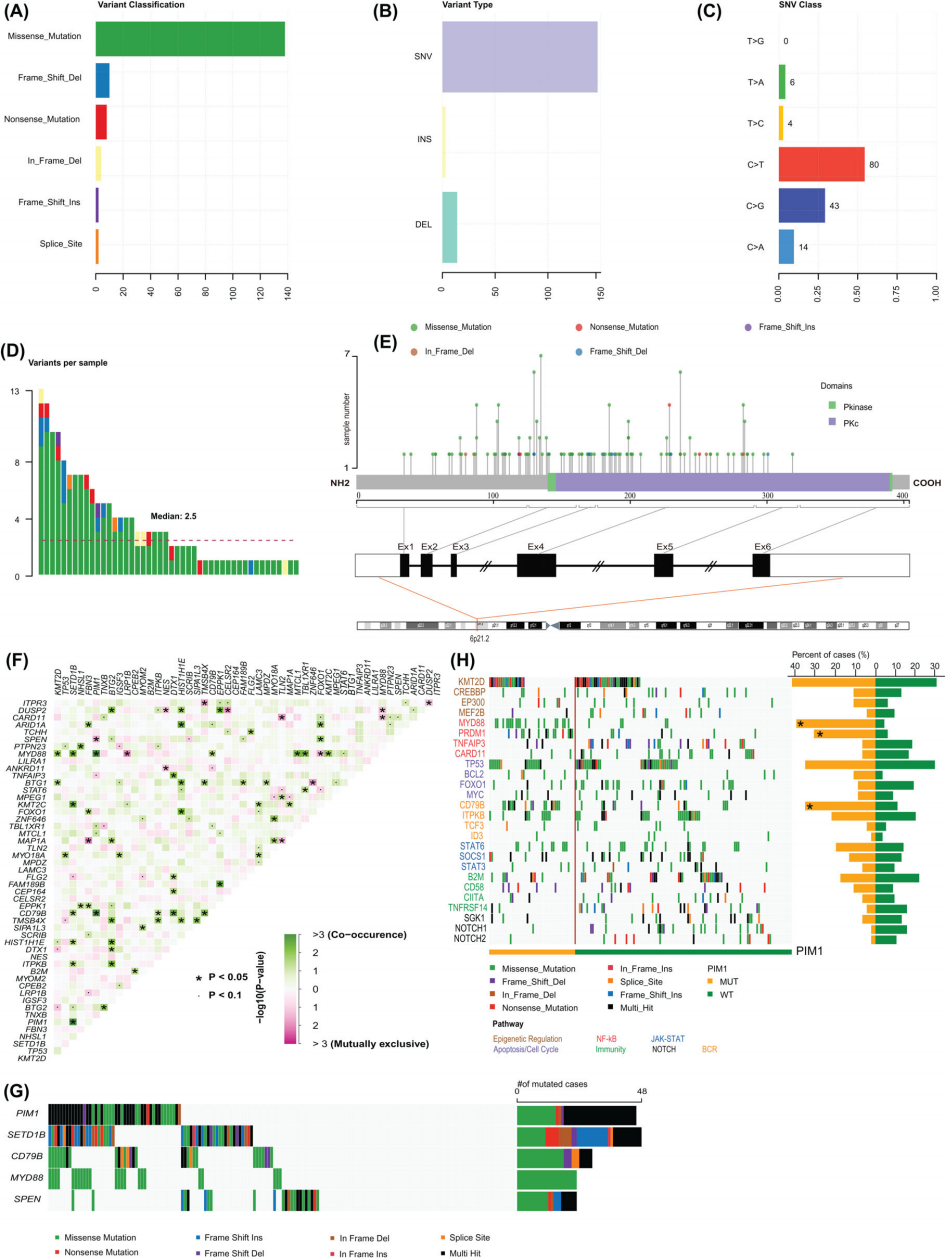
*PIM1* variants, structure, and interaction analysis of mutations. (A) The number of *PIM1* variant classifications detected. (B) Counts of *PIM1* variant types detected. (C) Summary of *PIM1* base substitutions. (D) Variants of *PIM1* per mutant patient. (E) Schematic representation of the domain structure and locations of the somatic mutations within *PIM1* observed. The lines represent the position of the mutations. (F) Co‐mutational and mutually exclusive patterns in gene pairs based on the top 50 most frequently mutated genes. The green squares represent co‐occurring mutations, and pink represent mutually exclusive mutations. The intensity of the color is correspondent to the ‐log10 (*p*‐value). (G) Significant examples of co‐occurring mutations (*PIM1* and *SETD1B*, *PIM1* and *CD79B*) and mutual exclusivity (*PIM1* and *SPEN*). (H) Overview of the distinct profiles of mutant *PIM1* and wild‐type *PIM1* diffuse large B‐cell lymphoma (DLBCL). Each column represents one sample, and each line represents one gene. Genes (in rows) are grouped according to their involvement in the signalling pathway with a color code and ordered according to their mutation frequencies within each group

Compared with wild‐type patients, those with mutations had significantly higher IPI scores (*p *= .031), especially in the high‐risk subgroup (17.4% vs. 4.3%), and were more likely to relapse (50% vs. 32%, *p *= .031); there was a trend toward a higher proportion in the non‐GCB subtype (52% vs. 39%) (Table S7). In particular, patients harbouring *PIM1* mutations more frequently had testis and/or CNS involvement (73% (8/11) vs. 25% (38/151), *p *= .001) (Figure S2). Of the 126 patients with available survival data, progression‐free survival (PFS) and overall survival (OS) were significantly shorter in the mutation group than in the wild‐type group (PFS, *p *= .022; OS, *p *= .0022), which was confirmed in the external validation cohort (*p *= .0022) (Figure 1B–D). In multivariate Cox analysis, *PIM1* mutation remained an independent unfavourable prognostic factor (*p *= .004) (Table S8). In short, *PIM1* mutations identify a molecular subgroup of DLBCL with inferior prognosis.

By using RNA sequencing, we first revealed that *PIM1* mutation led to a significantly higher level of gene expression (*p *< .001) (Figure 3A). Furthermore, several upregulated genes (*n* = 175) involved in the immune response (*IGLC6*, *IGLJ6*, *CLEC4C*), posttranslational modification (*ADPRHL1*, *NEURL1*), nuclear RNA export (*NXF3*), carcinogenesis (*WIF1*, *WNT9A*), and transcription factors (*HMX3, ZNF320*) (Figure 3B; Table S9) were enriched in patients with *PIM1* mutation. The markedly upregulated and downregulated genes are shown in Figure 3C. GO analysis results are depicted in Figure 3D (Table S10). Kyoto Encyclopedia of Genes and Genomes (KEGG) analysis revealed disorder of the tumour microenvironment (e.g., cytokine–cytokine receptor interaction, chemokine signalling, TNF signalling), JAK‐STAT and NF‐κB pathways in patients with *PIM1* mutation (Figure 3E). We then constructed a protein–protein interaction (PPI) network of five significant modules (Figure S3A,B), and the most significant module (Cluster 1, MCODE score = 11.286) was also mainly involved in the cellular response to chemokines, the TNF and the NF‐κB signalling pathway (Figure S3C,D).

**FIGURE 3:**
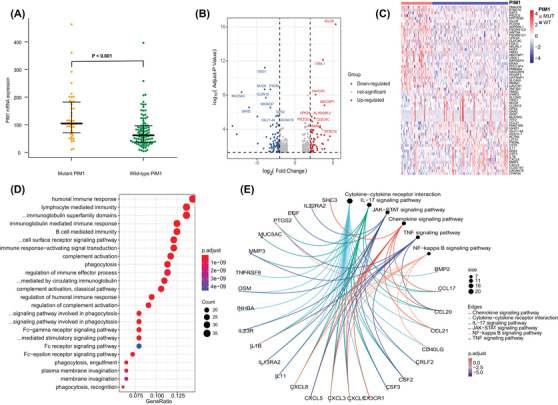
Biological function analysis mediated by mutant *PIM1* (MUT‐*PIM1*) compared to wild‐type *PIM1* (WT‐*PIM1*). (A) Comparison of *PIM1* gene expression between MUT‐*PIM1* and WT‐*PIM1*. (B) Volcano plot and (C) heat map (top 30 upregulated and top 30 downregulated genes with fold change ≥1.0 and *p* < .05) revealed different gene expression patterns between the MUT*‐PIM1* and WT‐*PIM1* groups. (D) GO plots of the differentially expressed genes (DEGs). (E) KEGG analysis results of the DEGs

Through multivariate analysis, three genes, *P2RY14*, *KRT80* and *OSM*, were identified as independent prognostic factors among 427 differentially expressed genes (DEGs) (Table S11). We then established the *PIM1* mutation‐related gene signature based on their expression levels (Table S12) and stratified patients into low‐ and high‐risk subgroups by the median risk score (Figure 4A), which showed independent prognostic significance (*p *= .002; Figure S4A,B). We found that high‐risk patients had significantly unfavourable OS (*p *= .0016) (Figure 4B) and PFS (*p *< .001) (Figure S4C,D). The areas under the curve (AUCs) suggested that the risk score had satisfactory sensitivity and specificity (Figure 4C). Similar results were obtained in the external validation cohort (Table S13, Figure 4D–F). Moreover, patients with high‐risk scores in both the age > 60 year and high IPI groups had significantly shorter OS and PFS, and also validated in the external cohort (Figure S5). There were 17 patients with *PIM1* mutations in the high‐risk group, and patients in this group had higher *PIM1* mutation rates (Figure S6). In particular, when *PIM1* mutation status was combined with the risk score, we found that patients with mutation and high risk had the poorest PFS (*p* = .0003) and OS (*p *< .0001) (Figure S7). Based on the GDSC database, patients with high risk scores exhibited higher sensitivity to some drugs targeting the immune microenvironment, including the TGFβ receptor inhibitors SB525334 (*p *< .0001) and the immunomodulator lenalidomide (*p *= .041), as well as the NF‐κB inhibitors parthenolide (*p *< .0001) and the JAK inhibitors ruxolitinib (*p *= .014)(Figure 4G). Other chemotherapeutic drugs are provided in Figure S8. Our findings suggest that the novel signature not only improves prognostic stratification but also provides personalized therapeutic decisions for patients with high risk.

**FIGURE 4:**
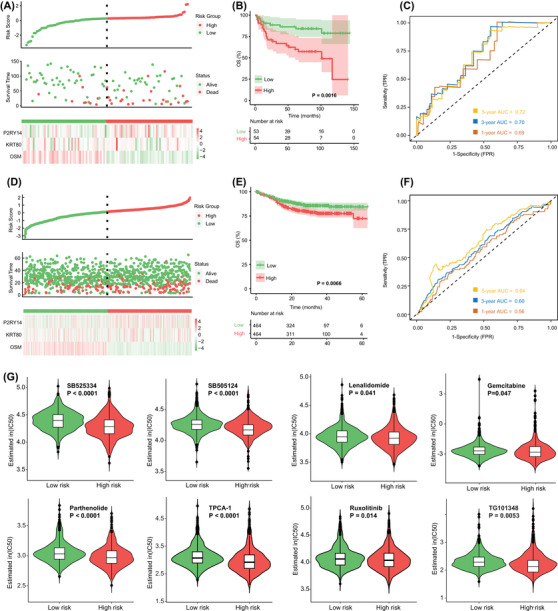
Identification and validation of the risk score based on the *PIM1* mutation‐related gene signature. (A) Risk scores distribution, survival status and gene expression heat map in our cohort. (B) Kaplan–Meier survival analysis of overall survival (OS) between different risk groups in our cohort. (C) Time‐dependent ROC analysis of 1‐, 3‐ and 5‐year OS in our cohort. (D) Risk scores distribution, survival status and gene expression heat map in the validation cohort. (E) Kaplan–Meier survival analysis of OS between different risk groups in the validation cohort. (F) Time‐dependent ROC analysis of 1‐, 3‐ and 5‐year OS in the validation cohort. (G) Estimated half‐maximal inhibitory concentration (IC50) value of each diffuse large B‐cell lymphoma (DLBCL) patients with low‐risk and high‐risk scores for anticancer drugs

In summary, our study reveals that *PIM1* mutation is involved in the pathogenesis of DLBCL, suggesting that detection of *PIM1* mutations with incorporation of our *PIM1* mutation‐related gene signature will be helpful for identifying DLBCL patients at high risk of progression and might provide predictive information for the design of personalized therapeutic strategies.

## CONFLICT OF INTEREST

The authors declare no conflict of interest.

## FUNDING INFORMATION

Natural Science Foundation of Tianjin; Grant Number: 19JCYBJC26500; National Natural Science Foundation of China, Grant Number: 81770213; Clinical Oncology Research Fund of CSCO, Grant Numbers: Y‐XD2019‐162 and Y‐Roche20192‐0097; National Human Genetic Resources Sharing Service Platform/Cancer Biobank of Tianjin Medical University Cancer Institute and Hospital, Grant Number: 2005DKA21300.

## PATIENT CONSENT STATEMENT

Written informed consent was obtained from all patients.

## Supporting information

Supplementary materials

**Fig. S1** Flowchart of analyses in the enrolled DLBCL patients in TMUCIH training cohort.
**Fig. S2** The involvement of testicular and/or central nervous system according to PIM1 mutation status. IP‐DLBCL, immune‐privileged site‐associated DLBCL; Non‐IP‐DLBCL, No immune‐privileged site‐associated DLBCL.
**Fig. S3 PPI network and module analysis based on the *PIM1* mutations‐related DEGs. a** PPI network of the 427 total DEGs. The color represents the degree of the nodes. **b** Five significant clusters of the PPI network based on the MCODE analysis. Cluster 1 is the most significant interactions module (MCODE score = 11.286). GO terms **(c)** and KEGG pathway analysis **(d)** based on the genes of cluster 1.
**Fig. S4** Clinical implications of the risk score based on the *PIM1* mutation‐related gene signature. Forrest plot of univariate (**a**) and multivariate (**b**) Cox regression analyses. Kaplan‐Meier survival analysis of PFS between different risk score subgroups based on the *PIM1* mutation‐related gene signature in our cohort (n = 107) (**c**) and validation cohort (n = 928) (**d**).
**Fig. S5** Kaplan‐Meier survival analysis between different risk groups in DLBCL patients. OS of patients with > 60 years subtype in our cohort **(a)** and the validation cohort **(e)**. OS of patients with IPI score 3–5 subtype in our cohort **(b)** and the validation cohort **(f)**. PFS of patients with > 60 years subtype in our cohort **(c)** and the validation cohort **(g)**. PFS of patients with IPI score 3–5 subtype in our cohort **(d)** and the validation cohort **(h)**.
**Fig. S6** Correlation between risk score and the status of *PIM1* mutations.
**Fig. S7** Kaplan‐Meier survival analysis of OS **(a)** and PFS **(b)** among patients with MUT&High risk, MUT&Low risk, WT&High risk and WT&Low risk group.
**Fig. S8** Estimated half‐maximal inhibitory concentration (IC50) value of each DLBCL patients with low‐risk and high‐risk scores for anticancer drugs.

**Supplemental Table S1**. Baseline clinical characteristics of 162 DLBCL patients.
**Supplementary Table S2**. The variant and individual‐level information of 283 genes in 162 patients.
**Supplementary Table S3**. One hundred and sixty‐four genetic alterations of PIM1 gene in 46 mutant DLBCL patients.
**Supplemental Table S4**. Functional impact of PIM1 mutations encoding protein kinases.
**Supplementary Table S5**. Co‐occurrence and mutually exclusive interaction analyses based on the top 50 most frequently mutated genes.
**Supplementary Table S6**. Overview of hot‐spot genes included in important signalling pathways in DLBCL.
**Supplementary Table S7**. Clinical Characteristics of Patients based on PIM1 Status.
**Supplementary Table S8**. Prognostic Factors affecting OS of DLBCL.
**Supplementary Table S9**. RNA‐seq analysis: list of 427 differentially expressed genes from comparison of PIM1 mutant and wild‐type DLBCL patients.
**Supplementary Table S10**. Seventy‐two items of the Gene Ontology enrichment analysis.
**Supplementary Table S11**. Results of univariate and multivariate Cox regression analyses based on the cluster 1–5 genes.
**Supplementary Table S12**. Risk score of each patient in the training DLBCL cohort (n = 107).
**Supplementary Table S13**. Risk score of each patient in the validation DLBCL cohort (n = 928).

## References

[1] Miao Y , Medeiros LJ , Li Y , Li J , Young KH . Genetic alterations and their clinical implications in DLBCL. Nat Rev Clin Oncol. 2019;16:634‐652. 31127191 10.1038/s41571-019-0225-1

[2] Trinh DL , Scott DW , Morin RD , et al. Analysis of FOXO1 mutations in diffuse large B‐cell lymphoma. Blood. 2013;121:3666‐3674. 23460611 10.1182/blood-2013-01-479865 PMC3643765

[3] Fernández‐Rodríguez C , Bellosillo B , García‐García M , et al. MYD88 (L265P) mutation is an independent prognostic factor for outcome in patients with diffuse large B‐cell lymphoma. Leukemia. 2014;28:2104‐2106. 24903481 10.1038/leu.2014.184

[4] Xu‐Monette ZY , Wu L , Visco C , et al. Mutational profile and prognostic significance of TP53 in diffuse large B‐cell lymphoma patients treated with R‐CHOP: report from an International DLBCL Rituximab‐CHOP Consortium Program Study. Blood. 2012;120:3986‐3996. 22955915 10.1182/blood-2012-05-433334 PMC3496956

[5] Pasqualucci L , Neumeister P , Goossens T , et al. Hypermutation of multiple proto‐oncogenes in B‐cell diffuse large‐cell lymphomas. Nature. 2001;412:341‐346. 11460166 10.1038/35085588

[6] Szydłowski M , Prochorec‐Sobieszek M , Szumera‐Ciećkiewicz A , et al. Expression of PIM kinases in Reed‐Sternberg cells fosters immune privilege and tumor cell survival in Hodgkin lymphoma. Blood. 2017;130:1418‐1429. 28698206 10.1182/blood-2017-01-760702

[7] De Smedt R , Morscio J , Reunes L , et al. Targeting cytokine‐ and therapy‐induced PIM1 activation in preclinical models of T‐cell acute lymphoblastic leukemia and lymphoma. Blood. 2020;135:1685‐1695. 32315407 10.1182/blood.2019003880

[8] Brasó‐Maristany F , Filosto S , Catchpole S , et al. PIM1 kinase regulates cell death, tumor growth and chemotherapy response in triple‐negative breast cancer. Nat Med. 2016;22:1303‐1313. 27775704 10.1038/nm.4198 PMC5552044

[9] Schmitz R , Wright GW , Huang DW , et al. Genetics and pathogenesis of diffuse large B‐cell lymphoma. N Engl J Med. 2018;378:1396‐1407. 29641966 10.1056/NEJMoa1801445 PMC6010183

[10] Chapuy B , Stewart C , Dunford AJ , et al. Molecular subtypes of diffuse large B cell lymphoma are associated with distinct pathogenic mechanisms and outcomes. Nat Med. 2018;24:679‐690. 29713087 10.1038/s41591-018-0016-8 PMC6613387

