# Supplementary material for: PIM1 genetic alterations associated with distinct molecular profiles, phenotypes and drug responses in diffuse large B‐cell lymphoma
Source: Clin Transl Med. 2022 Apr 12;12(4):e808. doi: 10.1002/ctm2.808 (PMC9005929; doi:10.1002/ctm2.808)
Supplement: Supplementary file 1 — Supplementary materials [file CTM2-12-e808-s001.docx]

Supplementary materials for

**PIM1 genetic alterations associated with distinct molecular profiles, phenotypes and drug responses in diffuse large B-cell lymphoma**

**Materials and methods**

**Patients**

***TMUCIH training cohort***

A total of 188 patients from 2008 to 2018 at Tianjin Medical University Cancer Institute and Hospital (TMUCIH, Tianjin, China) were collected were further confirmed centrally by two experienced pathologists independently according to the 2016 World Health Organization classification criteria^1^, and the COO classifications were determined by Hans algorithm^2^. Patients were excluded if they developed recurrences (n=14), or had serious complications (n=3) and were lost to follow-up (n=11). All remaining 162 patients received R-CHOP/CHOP-like regimen, of whom 126 who received at least 2 cycles of therapy were enrolled in the survival analysis. The study protocol was approved by the institutional review board of TMUCIH. All patients provided written informed consent.

***Public database validation cohorts***

Gene mutations, gene expression and survival of two independent validation cohorts were respectively collected from online exome sequencing database (Accession number EGA: EGAS00001002606, n=1001)^3^ and Gene Expression Omnibus (GEO) database (Accession number: GSE117556, n=928).

**Targeted sequencing**

One hundred and sixty-two fresh-frozen DLBCL biopsy samples were extracted using the DNeasy Tissue and Blood Kit ([Qiagen, Hilden, Germany](http://muchong.com/t-4942641-1)). DNA concentration was measured by Qubit^®^ DNA Assay Kit in Qubit^®^ 2.0 Flurometer (Life Technologies, CA, USA).

A total amount of 0.6 μg of genomic DNA per sample was used as input material for the DNA sample preparation. Sequencing libraries were generated using Agilent SureSelectXT Custom 0.5-2.9Mb kit (Agilent Technologies, CA, USA) following manufacturer’s recommendations and index codes were added to each sample. Briefly, fragmentation was carried out by hydrodynamic shearing system (Covaris, Massachusetts, USA) to generate 180-280bp fragments. After end-repaired and adenylated on the 3-ends, Illumina Paired-End (PE) adapters were ligated to DNA fragments to generate an indexed library using the SureSelect XT protocol (Agilent Technologies), which was quantified on an Agilent 2100 Bioanalyzer (Santa Clara, CA, USA). DNA fragments with ligated adapter molecules on both ends were selectively enriched in a PCR reaction. After PCR reaction, libraries hybridize with liquid phase with biotin labeled probe, then use magnetic beads with streptomycin to capture the target region. Captured libraries were enriched in a PCR reaction to add index tags to prepare for sequencing. Products were purified using AMPure XP system (Beckman Coulter, Beverly, USA) and quantified using the Agilent high sensitivity DNA assay on the Agilent Bioanalyzer 2100 system. The clustering of the index-coded samples was performed on a cBot Cluster Generation System using Hiseq PE Cluster Kit(Illumina) according to the manufacturer’s instructions. After cluster generation, the DNA libraries were sequenced on Illumina Hiseq platform and 150 bp paired-end reads were generated based on a 307 lymphoma-related gene panel. Sequence artifacts were discarded as follows: 1) Discard a paired reads if either one read contains adapter contamination (>10 nucleotides aligned to the adapter, allowing ≤ 10% mismatches); 2) Discard a paired reads if more than 10% of bases are uncertain in either one read; 3) Discard a paired reads if the proportion of low quality (Phred quality <5) bases is over 50% in either one read.

Valid sequencing data were then aligned to the Human Genome Reference Consortium build 37(GRCh37/hg19) by BWA (v0.7.12). Then, SAM tools, Picard (v1.87; <http://broadinstitute.github.io/picard/>) and Genome Analysis Toolkit (GATK) were used to sort BAM files and performing repeated marking, local realignment, and base quality recalibration. Single nucleotide variants (SNVs) were identified using the GATK Unified Genotyper and indels were determined using VarScan^4^. ANNOVAR package were used for annotation of all substitutions and indels. After annotation, we further filtered the mutations as follows: (1) synonymous; (2) the depth of the alternative allele was >5; and (3) with allele fraction <30%; (4) variants with <10 reads for SNPs and <10 reads for INDELs; (5) the impact of the mutations predicted by SnpEff was “high” or “moderate”. The effect of the detected missense variants on protein structure or function was analyzed with SIFT (<https://sift.bii.a-star.edu.sg>). The R package “maftools” was used to perform the mutation spectrum analysis.

**Transcriptome sequencing**

Total RNA was isolated using RNeasy Kit (Qiagen, Hilden, Germany) from fresh-frozen tumor tissue of 140 patients among the 162 DLBCL. RNA purity was checked using the NanoPhotometer® spectrophotometer (IMPLEN, CA, USA). RNA concentration and integrity was measured by Qubit® RNA Assay Kit (Life Technologies, CA, USA) and Bioanalyzer 2100 system (Agilent Technologies, CA, USA). A total amount of 3 μg RNA per sample was used as input material for the RNA sample preparations. Sequencing libraries were generated using NEBNext® UltraTM RNA Library Prep Kit for Illumina® (NEB, USA) following manufacturer’s recommendations and index codes were added to attribute sequences to each sample. Briefly, mRNA was purified from total RNA using poly-T oligo-attached magnetic beads. Fragmentation was carried out using divalent cations under elevated temperature in NEBNext First Strand Synthesis Reaction Buffer(5X). First strand cDNA was synthesized using random hexamer primer and M-MuLV Reverse Transcriptase (RNase H). Second strand cDNA synthesis was subsequently performed using DNA Polymerase I and RNase H. Remaining overhangs were converted into blunt ends via exonuclease/polymerase activities. After adenylation of 3’ ends of DNA fragments, NEBNext Adaptor with hairpin loop structure were ligated to prepare for hybridization. In order to select cDNA fragments of preferentially 250~300 bp in length, the library fragments were purified with AMPure XP system (Beckman Coulter, Beverly, USA). Then 3 μl USER Enzyme(NEB, USA) was used with size-selected, adaptor-ligated cDNA at 37°C for 15 min followed by 5 min at 95 °C before PCR. Then PCR was performed with Phusion High-Fidelity DNA polymerase, Universal PCR primers and Index (X) Primer. At last, PCR products were purified (AMPure XP system) and library quality was assessed on the Agilent Bioanalyzer 2100 system. The clustering of the index-coded samples was performed on a cBot Cluster Generation System using TruSeq PE Cluster Kit v3-cBot-HS (Illumia) according to the manufacturer’s instructions. After cluster generation, the library preparations were sequenced on an Illumina Hiseq platform and 125 bp/150 bp paired-end reads were generated. Clean data (clean reads) were obtained by removing reads containing adapter, reads containing ploy-N and low quality reads from raw data.

Reference genome and gene model annotation files were downloaded from genome website directly. Index of the reference genome was built using Hisat2 v2.0.5 and paired-end clean reads were aligned to the reference genome using Hisat2 v2.0.5. FeatureCounts v1.5.0-p3 was used to count the reads numbers mapped to each gene. And then FPKM of each gene was calculated based on the length of the gene and reads count mapped to this gene.

Differentially expressed genes (DEGs) were screened between the mutant *PIM1* group and the wild-type *PIM1* group using the DESeq2 R package (1.16.1)^5^ and the thresholds were defined as |log2-fold change (FC)| >1 and false discovery rate (FDR) < 0.05. *P*-values were adjusted based on Benjamini and Hochberg’s approach to control FDR. DEG analysis was then visualized by volcano plot and heatmap which were generated using the pheatmap R package.

**Gene functional and pathway enrichment analyses of DEGs**

Gene Ontology (GO) functional and Kyoto Encyclopedia of Genes and Genomes (KEGG) pathway analyses were conducted to annotate the functions of the candidate DEGs using the “enrichplot,” “clusterProfler,” “ggplot2” and “org.Hs.eg.db” functions of R software. The pathways with adjusted P-value < 0.05 were considered as significant enrichments for both types of functional enrichment analyses.

**Protein-protein interaction (PPI) network construction of DEGs**

Search Tool for the Retrieval of Interacting Genes (STRING) (http://string-db.org) (version 11.0)^6^ was utilized for predicting functional interpretation of protein interactions. The DEGs entered the STRING website with the minimum interaction score being 0.400 (medium confidence), with the protein nodes which disconnected with other proteins removed. Next, the PPI network was constructed using Cytoscape software (http://www.cytoscape.org) (version 3.7.1), and the top 10 hub genes were selected via a Cytoscape plugin (degree ranking of cytoHubba). Subsequently, molecular complex detection (MCODE) tool in Cytoscape was utilized to identifie highly interconnected clusters with thresholds of node score cutoff = 0.2, K-Core = 2, and degree cutoff = 2.

**Establishment and validation of a novel *PIM1* mutation-related gene signature**

Univariate and multivariate Cox regression was performed to describe the correlation between the DEGs and the survival of 107 DLBCL patients. Genes with P < 0.1 were thought to be statistically significant. Finally, a novel *PIM1* mutation-related gene signature was established and patients were classified into low- and high-risk groups using the median risk score as the cut-off point. The risk score for the *PIM1* mutation-related gene signature was calculated as follows:

Risk score =$\sum_{\boldsymbol{i=1}}^{\boldsymbol{n}} \boldsymbol{Coef}_{\boldsymbol{i}}\boldsymbol{*}\boldsymbol{x}_{\boldsymbol{i}}$

where $\boldsymbol{Coef}$ is the coefficient in the multivariate Cox analysis and $\boldsymbol{x}_{\boldsymbol{i}}$ is the Z-score-transformed relative expression value of each gene.

The GSE117556 data set were downloaded from the Gene Expression Omnibus (GEO) database (<https://www.ncbi.nlm.nih.gov/geo/>) with available gene expression profile and clinical information for external validation. A total of 928 patients with follow-up time and survival status information were obtained in the GEO cohort. The risk score for each patient in the GEO cohort was calculated using the same formula. The Kaplan-Meier (K-M) method was used to evaluate the prognosis between different risk groups. The time-dependent receiver operating characteristic (tROC) curves were generated to assess the discrimination ability of the gene signature as measured by the Area Under the ROC Curve (AUC). Univariate and multivariate Cox regression analyses were conducted to confirm this signature’s independent prognostic value.

**Prediction of response to drug therapies**

The Genomics of Drug Sensitivity in Cancer (GDSC) database was applied to predict the therapeutic response of each DLBCL patient between the low- and high-risk groups to common chemotherapeutic and targeted drugs using the R package “*pRRophetic*”. The drug sensitivity of each patient which was linked to detailed genomic information was estimated by the half maximal inhibitory concentration (IC50). Tenfold cross-validation was used to evaluated the accuracy of predicted IC50 value. Mann-Whitney-Wilcoxon Test was used to test whether IC50 distributions in low- and high-risk subgroups were identical.

**Statistical analysis**

Statistics were carried out using R (version 4.0.3) and SPSS 25.0 (Chicago, Illinois). Chi-square test was used to analyze the association of *PIM1* mutations with clinical and pathological factors. Fisher’s exact tests were used for co-occurrence or mutual exclusion of mutations in gene pairs. Kaplan-Meier curves and log-rank tests were used to evaluate the significant difference in prognosis between different subgroups. Pairwise comparisons were performed with the Wilcoxon rank sum test. All *p* values were two-sided, with *p* < 0.05 indicating statistical significance.

**References**

1. Swerdlow SH, Campo E, Pileri SA, Harris NL, Stein H, Siebert R, *et al.* The 2016 revision of the World Health Organization classification of lymphoid neoplasms. *Blood* 2016; **127**: 2375-2390.

2. Hans CP, Weisenburger DD, Greiner TC, Gascoyne RD, Delabie J, Ott G, *et al.* Confirmation of the molecular classification of diffuse large B-cell lymphoma by immunohistochemistry using a tissue microarray. *Blood* 2004; **103**: 275-282.

3. Reddy A, Zhang J, Davis NS, Moffitt AB, Love CL, Waldrop A, *et al.* Genetic and functional drivers of diffuse large B cell lymphoma. *Cell* 2017; **171**: 481-494. e15.

4. Saunders CT, Wong WS, Swamy S, Becq J, Murray LJ, Cheetham RK*.* Strelka: accurate somatic small-variant calling from sequenced tumor-normal sample pairs. *Bioinformatics* 2012; **28**: 1811-1817.

5. Love MI, Huber W, Anders S. Moderated estimation of fold change and dispersion for RNA-seq data with DESeq2. *Genome Biol* 2014; **15**: 550.

6. Szklarczyk D, Gable AL, Lyon D, Junge A, Wyder S, Huerta-Cepas J, *et al.* STRING v11: protein-protein association networks with increased coverage, supporting functional discovery in genome-wide experimental datasets. *Nucleic Acids Res* 2019; **47**: D607-D613.
