# Supplementary material for: PIM1 genetic alterations associated with distinct molecular profiles, phenotypes and drug responses in diffuse large B‐cell lymphoma
Source: Clin Transl Med. 2022 Apr 12;12(4):e808. doi: 10.1002/ctm2.808 (PMC9005929; doi:10.1002/ctm2.808)
Supplement: Supplementary file 2 — Fig. S1 Flowchart of analyses in the enrolled DLBCL patients in TMUCIH training cohort. Fig. S2 The involvement of testicular and/or central nervous system according to PIM1 mutation status. IP‐DLBCL, immune‐privileged site‐associated DLBCL; Non‐IP‐DLBCL, No immune‐privileged site‐associated DLBCL. Fig. S3 PPI network and module analysis based on the PIM1 mutations‐related DEGs. a PPI network of the 427 total DEGs. The color represents the degree of the nodes. b Five significant clusters of the PPI network based on the MCODE analysis. Cluster 1 is the most significant interactions module (MCODE score = 11.286). GO terms (c) and KEGG pathway analysis (d) based on the genes of cluster 1. Fig. S4 Clinical implications of the risk score based on the PIM1 mutation‐related gene signature. Forrest plot of univariate (a) and multivariate (b) Cox regression analyses. Kaplan‐Meier survival analysis of PFS between different risk score subgroups based on the PIM1 mutation‐related gene signature in our cohort (n = 107) (c) and validation cohort (n = 928) (d). Fig. S5 Kaplan‐Meier survival analysis between different risk groups in DLBCL patients. OS of patients with > 60 years subtype in our cohort (a) and the validation cohort (e). OS of patients with IPI score 3–5 subtype in our cohort (b) and the validation cohort (f). PFS of patients with > 60 years subtype in our cohort (c) and the validation cohort (g). PFS of patients with IPI score 3–5 subtype in our cohort (d) and the validation cohort (h). Fig. S6 Correlation between risk score and the status of PIM1 mutations. Fig. S7 Kaplan‐Meier survival analysis of OS (a) and PFS (b) among patients with MUT&High risk, MUT&Low risk, WT&High risk and WT&Low risk group. Fig. S8 Estimated half‐maximal inhibitory concentration (IC50) value of each DLBCL patients with low‐risk and high‐risk scores for anticancer drugs. [file CTM2-12-e808-s002.docx]

Supplementary Figures for

**PIM1 genetic alterations associated with distinct molecular profiles, phenotypes and drug responses in diffuse large B-cell lymphoma**

**This file includes:**

Supplementary Figures S1 to S8


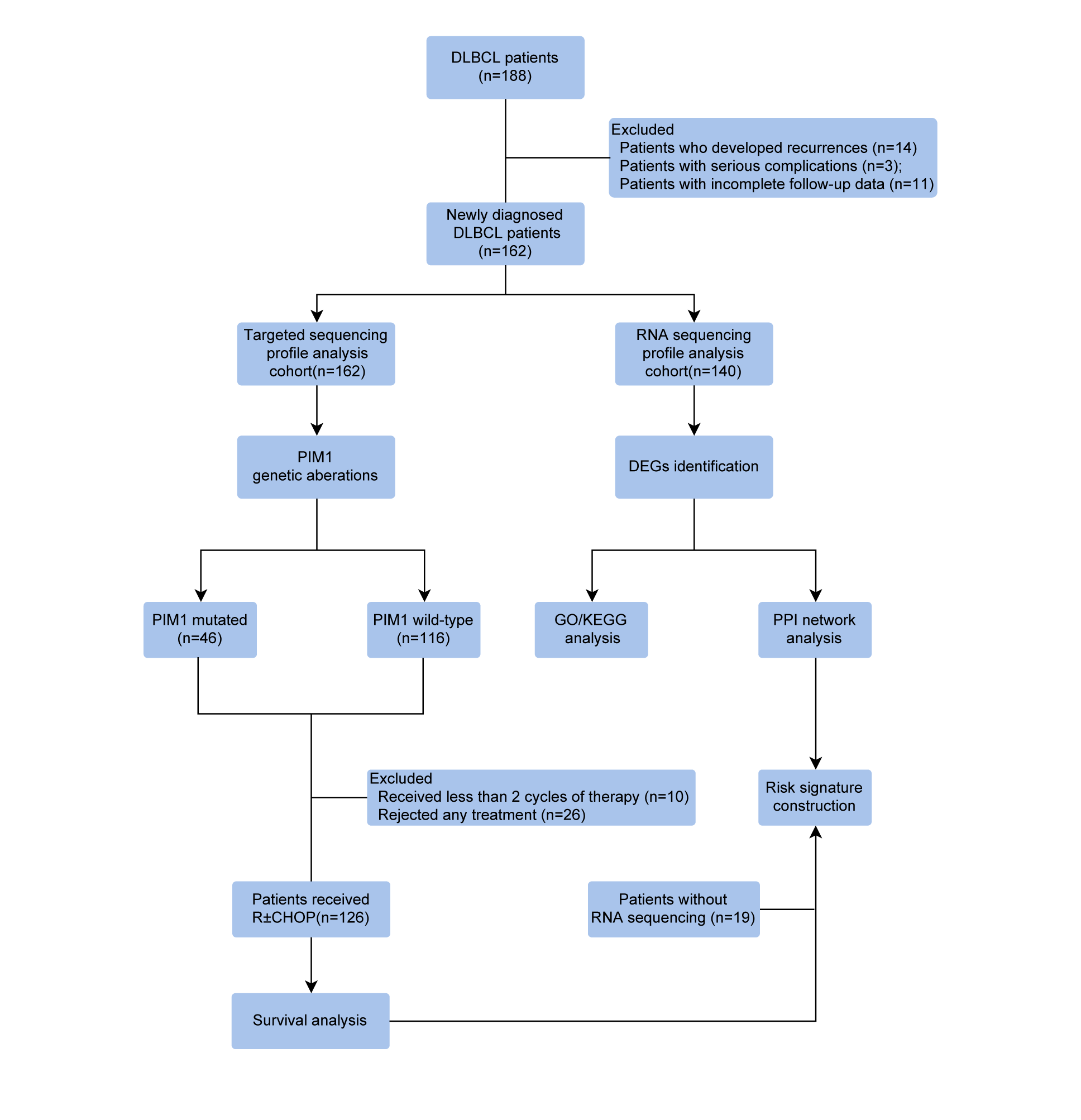


**Figure S1.** Flowchart of analyses in the enrolled DLBCL patients in TMUCIH training cohort.


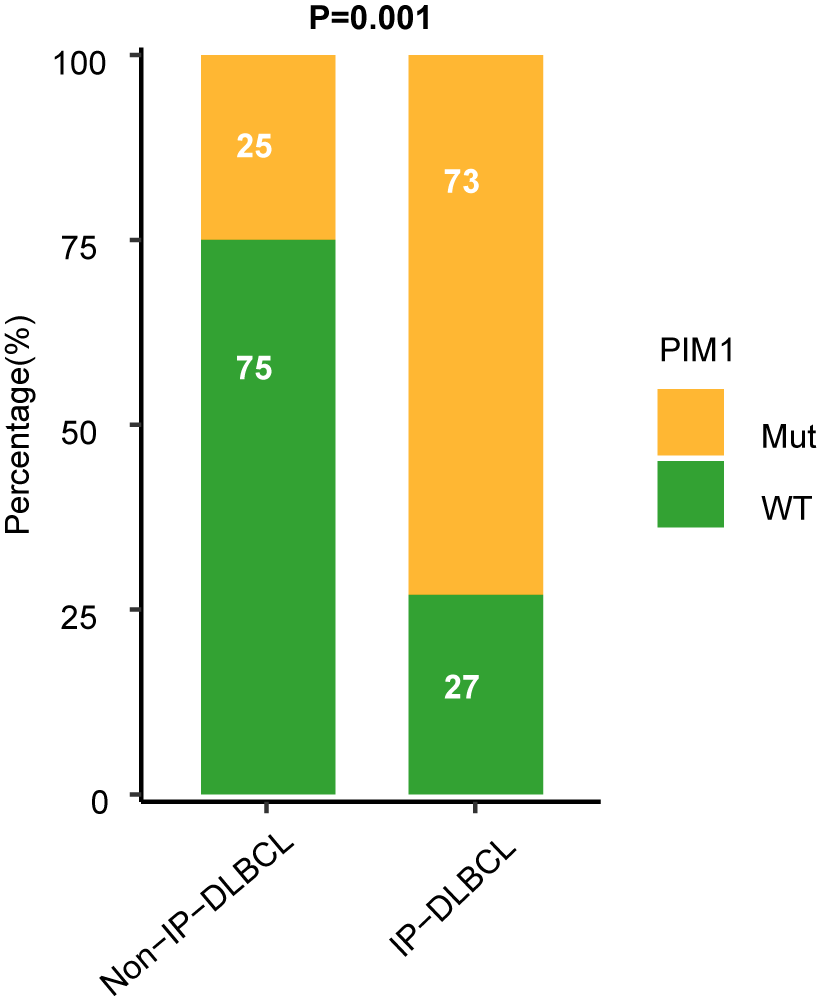


**Figure S2.** The involvement of testicular and/or central nervous system according to PIM1 mutation status. IP-DLBCL, immune-privileged site-associated DLBCL; Non-IP-DLBCL, No immune-privileged site-associated DLBCL.


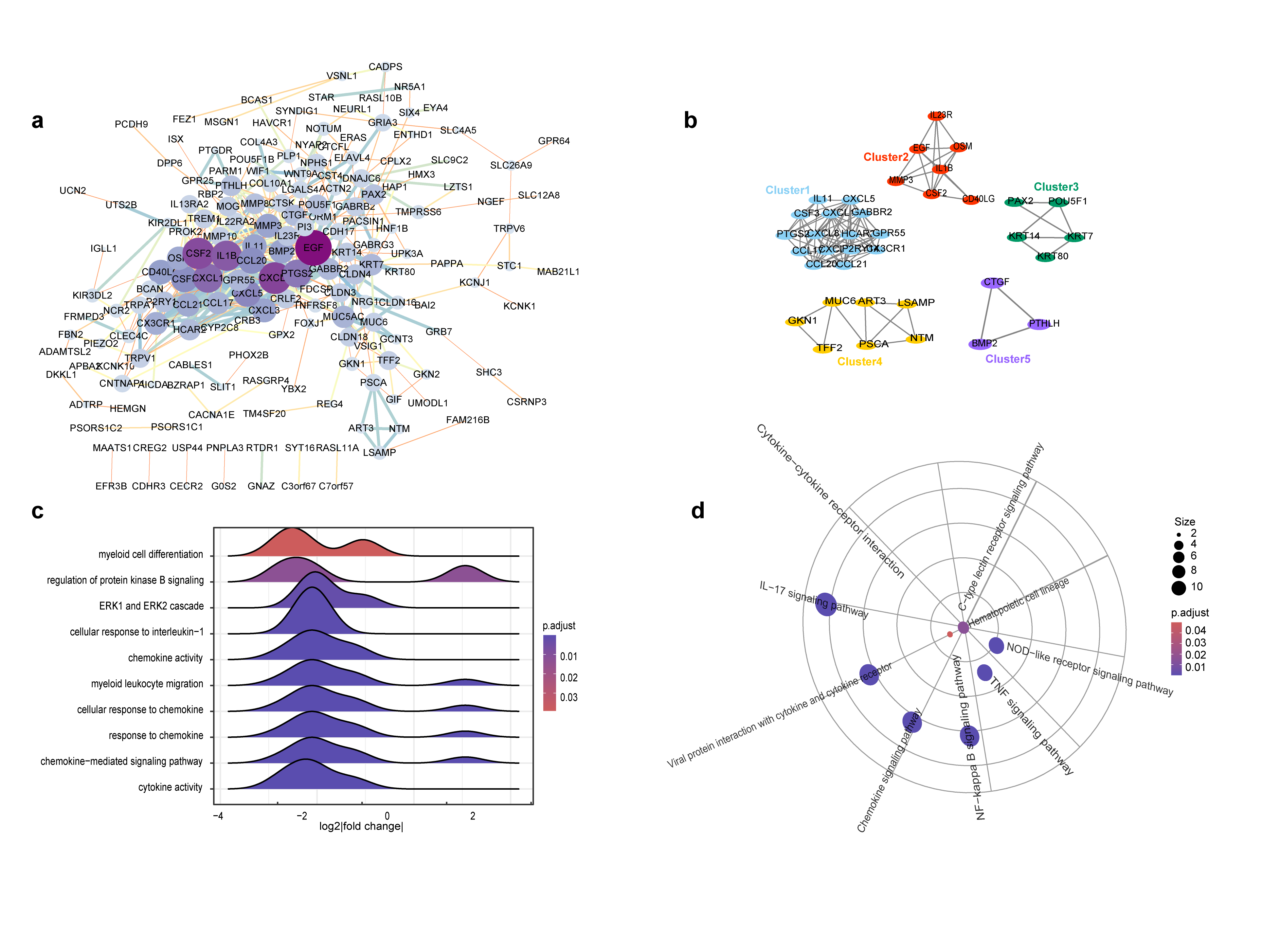


**Figure S3. PPI network and module analysis based on the *PIM1* mutations-related DEGs.** **a** PPI network of the 427 total DEGs. The color represents the degree of the nodes. **b** Five significant clusters of the PPI network based on the MCODE analysis. Cluster 1 is the most significant interactions module (MCODE score = 11.286). GO terms **(c)** and KEGG pathway analysis **(d)** based on the genes of cluster 1.


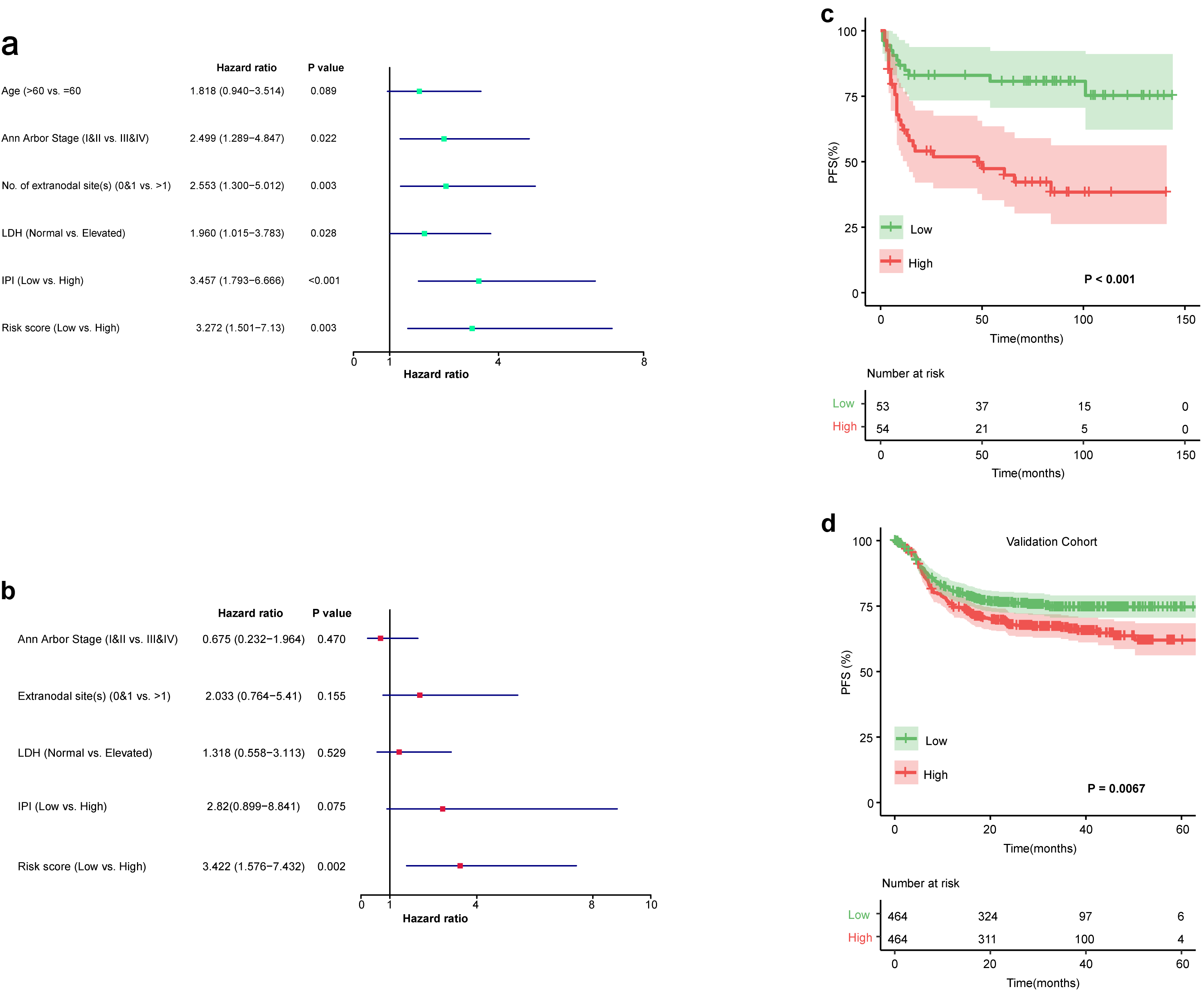


**Figure S4.** Clinical implications of the risk score based on the *PIM1* mutation-related gene signature. Forrest plot of univariate (**a**) and multivariate (**b**) Cox regression analyses. Kaplan-Meier survival analysis of PFS between different risk score subgroups based on the *PIM1* mutation-related gene signature in our cohort (n = 107) (**c**) and validation cohort (n = 928) (**d**).


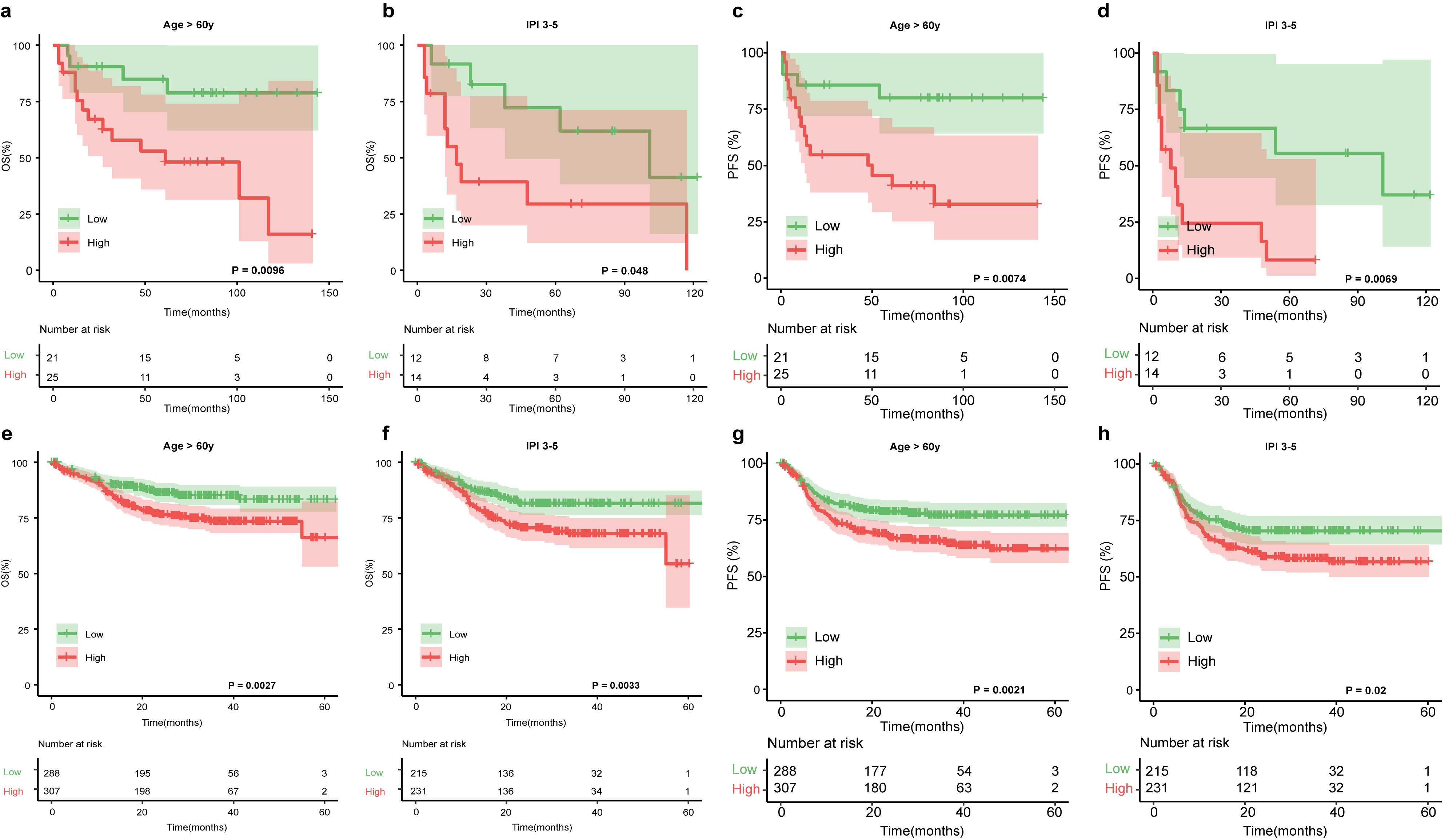


**Figure S5.** Kaplan-Meier survival analysis between different risk groups in DLBCL patients. OS of patients with > 60 years subtype in our cohort **(a)** and the validation cohort **(e)**. OS of patients with IPI score 3-5 subtype in our cohort **(b)** and the validation cohort **(f)**. PFS of patients with > 60 years subtype in our cohort **(c)** and the validation cohort **(g)**. PFS of patients with IPI score 3-5 subtype in our cohort **(d)** and the validation cohort **(h)**.

**
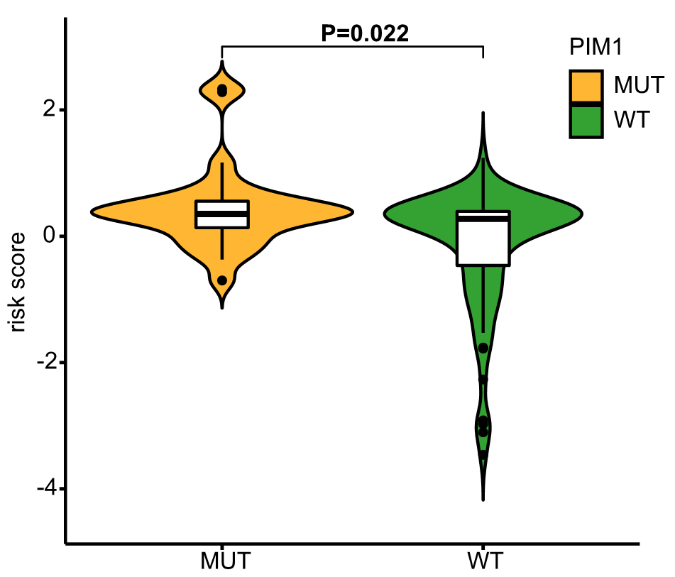
**

**Figure S6.** Correlation between risk score and the status of *PIM1* mutations.

**
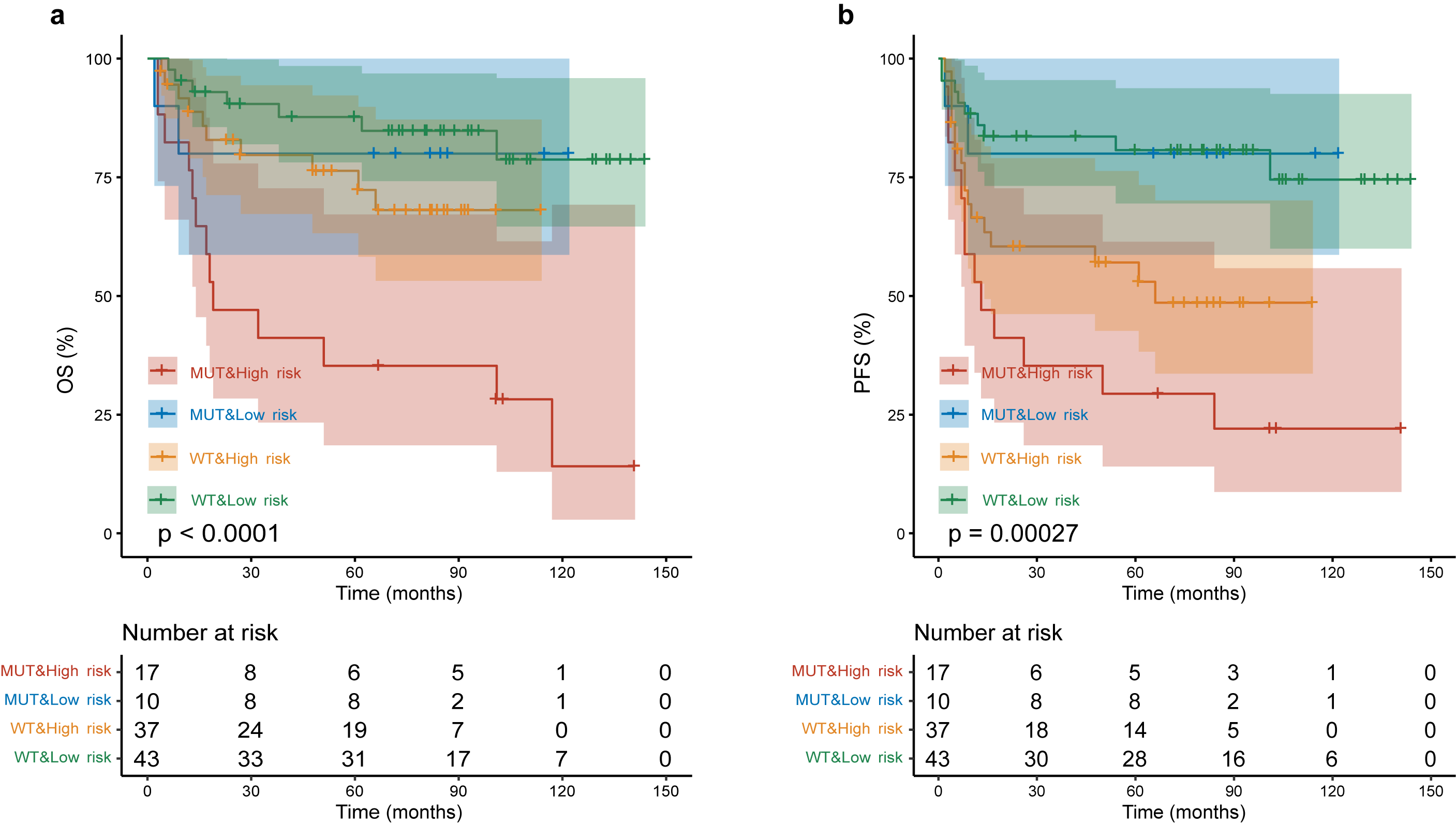
**

**Figure S7.** Kaplan-Meier survival analysis of OS **(a)** and PFS **(b)** among patients with MUT&High risk, MUT&Low risk, WT&High risk and WT&Low risk group.


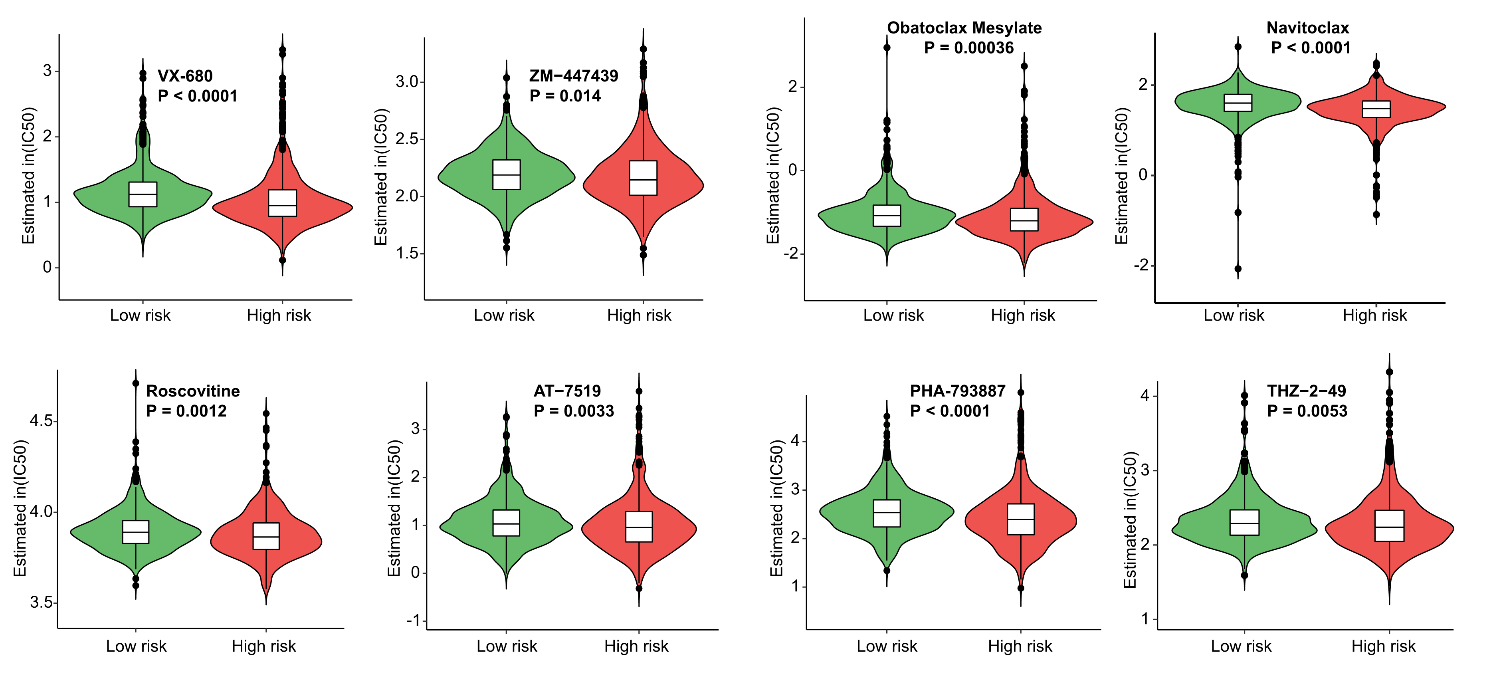


**Figure S8.** Estimated half-maximal inhibitory concentration (IC50) value of each DLBCL patients with low-risk and high-risk scores for anticancer drugs.
